# A new class of lightweight, stainless steels with ultra-high strength and large ductility

**DOI:** 10.1038/s41598-020-69177-7

**Published:** 2020-07-22

**Authors:** Joonoh Moon, Heon-Young Ha, Kyeong-Won Kim, Seong-Jun Park, Tae-Ho Lee, Sung-Dae Kim, Jae Hoon Jang, Hyo-Haeng Jo, Hyun-Uk Hong, Bong Ho Lee, Young-Joo Lee, Changhee Lee, Dong-Woo Suh, Heung Nam Han, Dierk Raabe, Chang-Hoon Lee

**Affiliations:** 1 Steel Department, Advanced Metals Division, Korea Institute of Materials Science, 797 Changwondae-ro, Seongsan-gu, Changwon, Gyeongnam 51508 Republic of Korea; 2 Department of Materials Science and Engineering, Changwon National University, 20 Changwondaehak-ro, Uichang-gu, Changwon, Gyeongnam 51140 Republic of Korea; 3 Center for Core Research Facilities, Daegu Gyeongbuk Institute of Science and Technology, 333 Hyeonpung-eup, Dalseong-gun, Daegu, 42988 Republic of Korea; 4 Advanced Metallic Materials Research Group, Research Institute of Industrial Science and Technology, 67 Cheongam-ro, Nam-gu, Pohang, Gyeongbuk 37673 Republic of Korea; 5 Division of Materials Science and Engineering, Hanyang University, Seoul, 04763 Republic of Korea; 6 Graduate Institute of Ferrous Technology, Pohang University of Science and Technology, 77 Cheongam-ro, Nam-gu, Gyeongbuk, 37673 Republic of Korea; 7 Department of Materials Science and Engineering and Research Institute of Advanced Materials, Seoul National University, 1 Gwanak-ro, Gwanak-gu, Seoul, 08826 Republic of Korea; 8 Max-Planck-Institut für Eisenforschung, Max-Planck-Straβe 1, 40237 Düsseldorf, Germany

**Keywords:** Metals and alloys, Mechanical properties

## Abstract

Steel is the global backbone material of industrialized societies, with more than 1.8 billion tons produced per year. However, steel-containing structures decay due to corrosion, destroying annually 3.4% (2.5 trillion US$) of the global gross domestic product. Besides this huge loss in value, a solution to the corrosion problem at minimum environmental impact would also leverage enhanced product longevity, providing an immense contribution to sustainability. Here, we report a leap forward toward this aim through the development of a new family of low-density stainless steels with ultra-high strength (> 1 GPa) and high ductility (> 35%). The alloys are based on the Fe–(20–30)Mn–(11.5–12.0)Al–1.5C–5Cr (wt%) system and are strengthened by dispersions of nano-sized Fe_3_AlC-type κ-carbide. The alloying with Cr enhances the ductility without sacrificing strength, by suppressing the precipitation of κ-carbide and thus stabilizing the austenite matrix. The formation of a protective Al-rich oxide film on the surface lends the alloys outstanding resistance to pitting corrosion similar to ferritic stainless steels. The new alloy class has thus the potential to replace commercial stainless steels as it has much higher strength at similar formability, 17% lower mass density and lower environmental impact, qualifying it for demanding lightweight, corrosion resistant, high-strength structural parts.

## Introduction

Corrosion is by far the most severe phenomenon limiting the longevity and integrity of metal products. For steels, as the leading engineering material class, this challenge was first tackled at the end of the nineteenth century, leading to the development of stainless steels. These are indispensable materials used for many safety–critical parts in chemical industries, power plants, transportation, home appliances, food industry and kitchen utensils owing to their excellent corrosion resistance and good mechanical properties^1–6^. For achieving their corrosion-resistant characteristics, typical stainless steels based on the Fe–Cr, Fe–Cr–C, and Fe–Cr–Ni systems must contain a minimum of 10.5 wt% Cr^7,8^. Irrespective of their great success the further use and development of stainless steels has already decades ago reached limits set by their high mass density of about 7.9 g/cm^3^ and the key ingredients Cr and Ni which are expensive and associated with substantial environmental burdens when mined and synthesized.

Particularly the first aspect, i.e. weight reduction, is important for improving the efficiencies of energy conversion systems and secures the safety of metal-made infrastructures^9–11^. Therefore, the challenge for stainless steels is to reduce their density while maintaining their corrosion-resistant characteristics and mechanical properties, all achieved at lower environmental impact, realized through the (partial) replacement of Cr and Ni^12^. Here, we solve these two long standing key problems with a new lightweight stainless design concept, based on the austenitic alloy system Fe–Mn–Al–C. This quaternary system has been investigated before with respect to weight reduced alloy design for structural components^13–20^. Al does not only effectively reduce the density of steel, but can also increase its strength through the formation of nano-sized κ-carbides^13^. With increasing Al content, Fe–Mn–Al–C based lightweight steels generally achieve excellent specific strengths through the combination of low mass density and high strain hardening^10^. However, excessive addition of Al leads to the formation of coarse intergranular precipitation of κ-carbide^21,22^, leading to an abrupt loss of ductility. In addition, an increase in the fraction of κ-carbide with increasing Al content makes the austenite matrix unstable, leading to the formation of ferrite at the grain boundaries^23^. As a result, excessive Al addition to Fe–Mn–Al–C based lightweight steels can promote formation of microstructural galvanic elements, thereby degrading the corrosion properties, despite the positive role that Al atoms play in producing a protective passive layer on the surface. These aspects have posed a critical limit to the wider application of high-strength lightweight steels in structural components. Several attempts have been made to substitute stainless steel with Fe–Mn–Al–C alloys^24,25^, however, conventional Fe–Mn–Al–C alloys showed inferior corrosion resistance compared to Cr- and Ni-containing stainless steels owing to their insufficient Al content (< 10 wt%), required to form a dense Al_2_O_3_ passive film.

Here, we report the design of a new family of ultra-low density, austenitic lightweight steels with excellent mechanical properties, high resistance to pitting corrosion and reduced environmental impact. We refer to this alloy class as lightweight stainless steel (LWSS). In order to overcome the limitations of conventional Fe–Mn–Al–C based austenitic lightweight steels explained above and for achieving high corrosion resistance together with a balance of high strength and ductility, we aimed to achieve a homogeneous microstructure under the condition of high Al content (> 11.5 wt%) through adequate alloy adjustment. To this end we utilized a minor blending with Cr, which suppresses the precipitation of inter-/intragranular κ-carbides, thereby contributing a homogeneous austenite matrix with minimal secondary phase formation. With this alloy adjustment LWSS exhibits the virtues of both lightweight steel and stainless steel, with ultra-high strength (> 1GPa), high elongation (> 35%), and significant density reduction (> 17%) compared to pure iron. It also provides resistance to pitting corrosion comparable to that of ferritic stainless steels and has low environmental impact as it uses no Ni and only a low Cr content.

## Results and discussion

**Figure 1:**
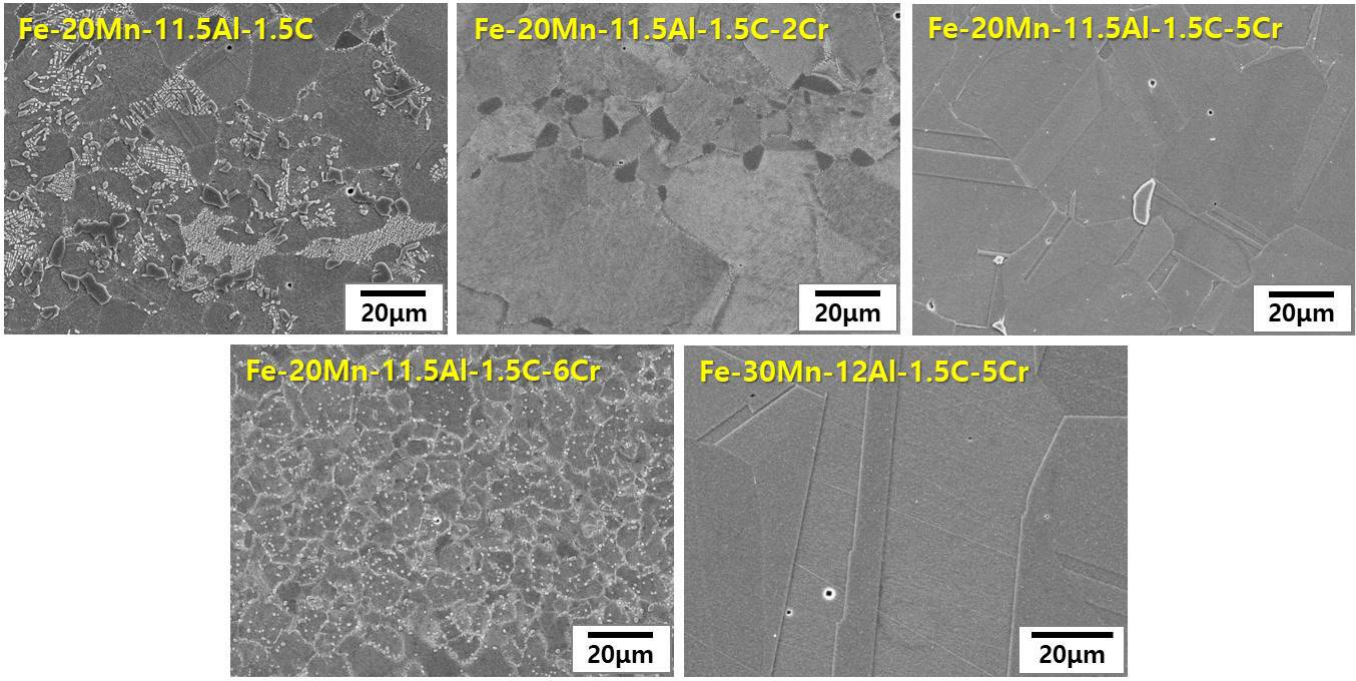
Change in the microstructure of Fe–(20–30)Mn–(11.5–12.0)Al–1.5C based alloys with increasing Cr content.

**Figure 2:**
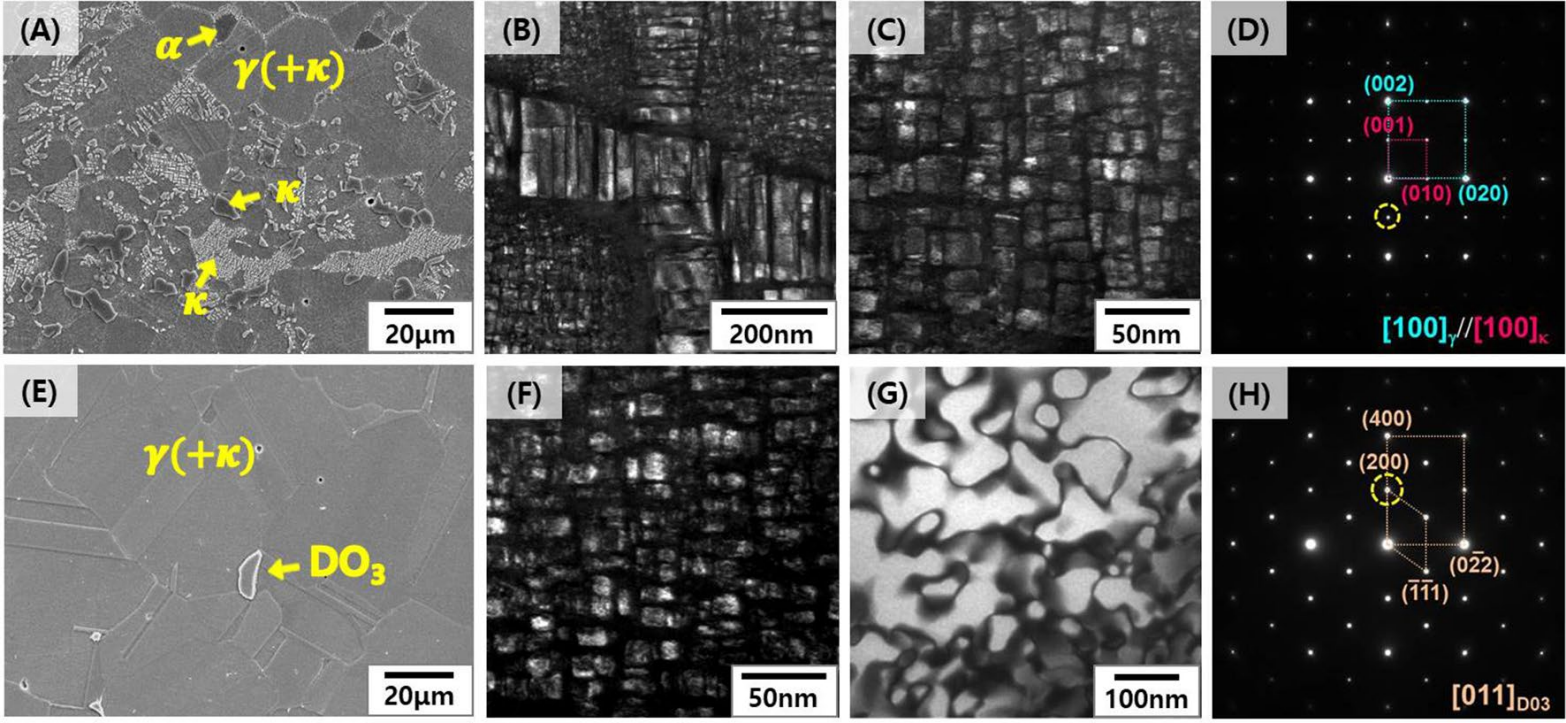
Microstructures of the solution-treated samples for 2 h at 1,050°C: (**A**) SEM image of the LWS1 alloy (Fe–20Mn–12Al–1.5C), (**B**,**C**) dark-field TEM images of coarse intergranular κ-carbide and nano-size κ-carbide within the austenite matrix, respectively; (**D**) selected area diffraction (SAD) pattern of the κ-carbide; (**E**) SEM image of the LWSS1 alloy (Fe–20Mn–11.5Al–1.5C–5Cr); (**F**,**G**) dark-field TEM images of coarse nano-size κ-carbide within the austenite and DO_3_ phase, respectively; (**H**) SAD pattern of the DO_3_ phase.

**Figure 3:**
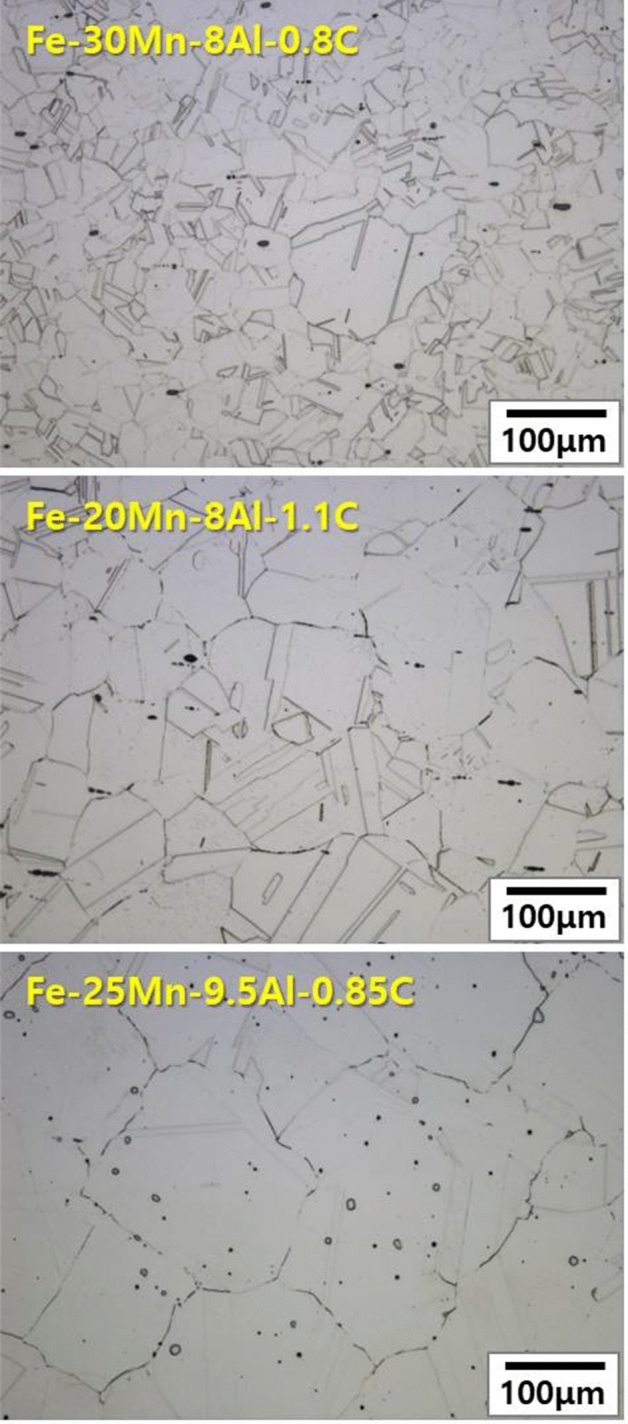
Optical microstructures of conventional lightweight steels.

Fe–Mn–Al–C based lightweight steels (LWS) with an austenite matrix were selected as starting point for the development of these novel lightweight stainless steels due to their excellent balance of strength and ductility at low mass density^13^. Also, Al aids the formation of a protective oxide layer at the steel’s surface, improving its resistance to galvanic corrosion in aqueous environments. More specific, previous work^26–28^ showed that the addition of Al to Fe–Mn–Al based alloys led to the formation of Al-bearing passive oxide films. Established austenitic lightweight steels mostly fall in the composition range Fe–(20–30)Mn–(8–12)Al–(0.8–1.5)C (all in weight %; chemical compositions for all reference alloys are in Table 1). Generally, the state of the matrix phase of Fe–Mn–Al–C alloys depends on the content of alloying elements; i.e., if large amounts of Al, a ferrite stabilizer, are added to achieve significant density reduction, the content of austenite stabilizing elements such as C and Mn must be increased to retain austenite. However, increasing the Al and C content leads to the formation of brittle phases, such as intergranular κ-carbides and FeAl-type B2^10,21^. Therefore, we explored a new alloy design approach for an ultra-low density lightweight steel with high Al content (11.5–12 wt%), which yields a stable austenite matrix without formation of brittle precipitates. For this purpose, we investigated the effect of Cr addition to a group of Fe–(20–30)Mn–(11.5–12.0)Al–1.5C (wt.%) alloys (Fig. 1) and found that a Cr content of 5 wt% is on the one hand stabilizing the austenite matrix (LWSS in Table 1) and on the other hand aiding the formation of a dense protective oxide layer (Fe–20Mn–11.5Al–1.5C–5Cr alloy and Fe–30Mn–12Al–15C–5Cr alloy). Figure 2A,E show microstructures of alloys LWS1 (the conventional lightweight steel) and LWSS1 (the new lightweight stainless steel), respectively, after solution treatment for 2 h at 1,050°C, revealed by scanning electron microscopy (SEM). Figure 2A shows that alloy LWS1 (Fe–20Mn–12Al–1.5C alloy) has a complex microstructure with an austenite (γ) matrix strengthened by fine ordered κ-carbide (Fig. 2B), coarse intergranular κ-carbide (Fig. 2C), and a small fraction of ferrite (α), as revealed by transmission electron microscopy (TEM) and selected area diffraction pattern (SADP) analyses (Fig. 2D). This microstructure is due to the high Al (11.9 wt%) and C (1.45 wt%) content. Alloys LWS2-4 containing relatively low Al (7.9–9.5 wt%) and C (0.8–1.1 wt%) contents have an austenite matrix, devoid of precipitates, Fig. 3. In contrast, the addition of larger amounts of Al and C into the base alloy LWS1 promotes formation of precipitation of intergranular κ-carbides, rendering the austenite matrix unstable and resulting in the formation of a secondary ferrite phase.

**Table 1:** Chemical compositions of the conventional lightweight steels and the lightweight stainless steels developed in this investigation.

Alloys		Chemical composition, wt%					
		C	Mn	Al	Ni	Cr	Fe
Conventional austenitic lightweight steels (LWS)	LWS1 (Fe–20Mn–12Al–1.5C)	1.45	19.10	11.90	–	–	Bal
	LWS2 (Fe–30Mn–8Al–0.8C)	0.81	28.51	7.95	–	–	Bal
	LWS3 (Fe–20Mn–8Al–1.1C)	1.12	19.57	8.17	–	–	Bal
	LWS4 (Fe–25Mn–9.5Al–0.85C)	0.84	24.80	9.45	5.07	–	Bal
Lightweight stainless steels (LWSS)	LWSS1 (Fe–20Mn–11.5Al–1.5C–5Cr)	1.43	19.99	11.47	–	4.88	Bal
	LWSS2 (Fe–30Mn–12Al–1.5C–5Cr)	1.48	29.05	12.01	–	5.13	Bal

**Figure 4:**
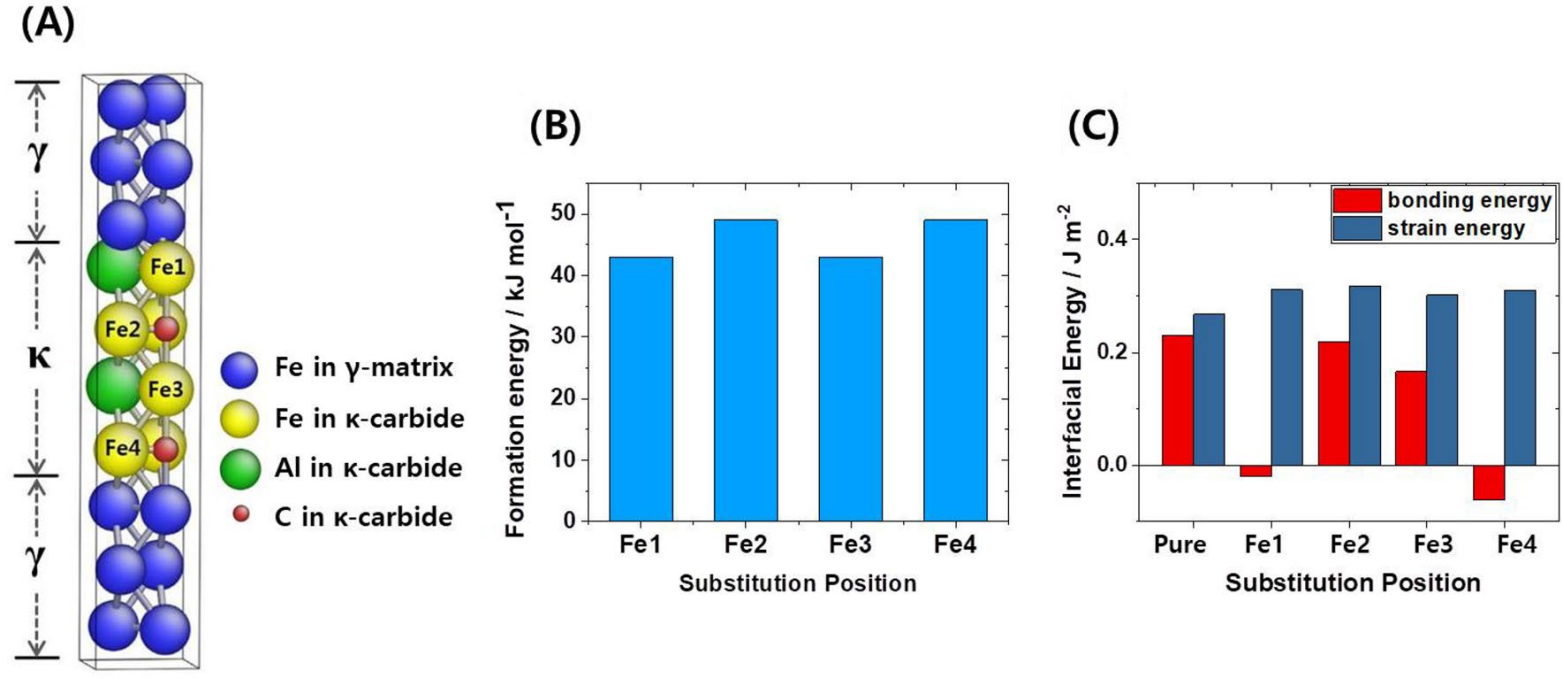
Results of first-principles calculations for the κ-carbide precipitation. (**A**) Unit cell showing the γ-matrix/κ-carbide interface structure, (**B**) calculated formation energies of κ-carbide, and (**C**) the calculated interfacial energies. The bars labeled Fe1, Fe2, Fe3, and Fe4 indicate the structures in which Fe atoms were replaced by Cr at the positions marked in the unit cell in Fig. 2A.

In this context Moon et al.^29^ had shown that the driving force for the precipitation of κ-carbide in such lightweight alloys decreases when adding Cr. We confirm this finding here through first-principles calculations^30–34^. For this purpose we simulated the system in terms of a supercell containing an austenitic unit cell with 12 Fe atoms and a coherent κ-carbide in the middle^35^. As shown in Fig. 4A, the substitution of Fe by Cr at four of the Fe atomic positions (Fe1, Fe2, Fe3, and Fe4) was considered in the simulations. Figure 4B shows the increase in formation energy for the resulting Fe_2_CrAlC structure, compared to the Fe_3_AlC reference structure for a system devoid of Cr. Figure 4B shows that the formation energies of the Fe_2_CrAlC structure variants are indeed at least 43 kJ/mol above those of the Fe_3_AlC structure. Figure 4C shows the interfacial energy results consisting of the chemical bonding and strain energies across the interface. The strain energy contribution to the interfacial energy for the case with the four Cr substitution positions is higher than that for Fe_3_AlC. These results thus show that the addition of Cr indeed suppresses the precipitation of κ-carbide in terms of both its formation energy and the strain energy at the interface^36^. Compared to the LWS1 reference alloy with Fe–20Mn–12Al–1.5C (wt.%), the LWSS1 alloy containing 5 wt% Cr has a microstructure consisting of an austenite matrix with a small fraction of DO_3_ phase, *i.e.* ordered phase with a body-centered cubic structure, Fig. 2E. The measured fraction of DO_3_ in the new lightweight stainless alloy was very small (0.8 vol.%). Also, we observe that the addition of 5 wt% Cr has entirely eliminated any harmful intergranular κ-carbides, in good agreement with the simulations, Fig. 4A–C. Obviously the addition of Cr also suppressed the precipitation of fine intragranular κ-carbides within the austenite matrix, i.e*.*, the fraction and mean size of κ-carbides within the austenite matrix decreased with an addition of 5 wt% Cr, as shown in Fig. 2C,F. Yet, the austenite is still strengthened by fine ordered κ-carbides (Fig. 2F), and the small DO_3_ phase (Fig. 2G,H)^34,37^.

**Figure 5:**
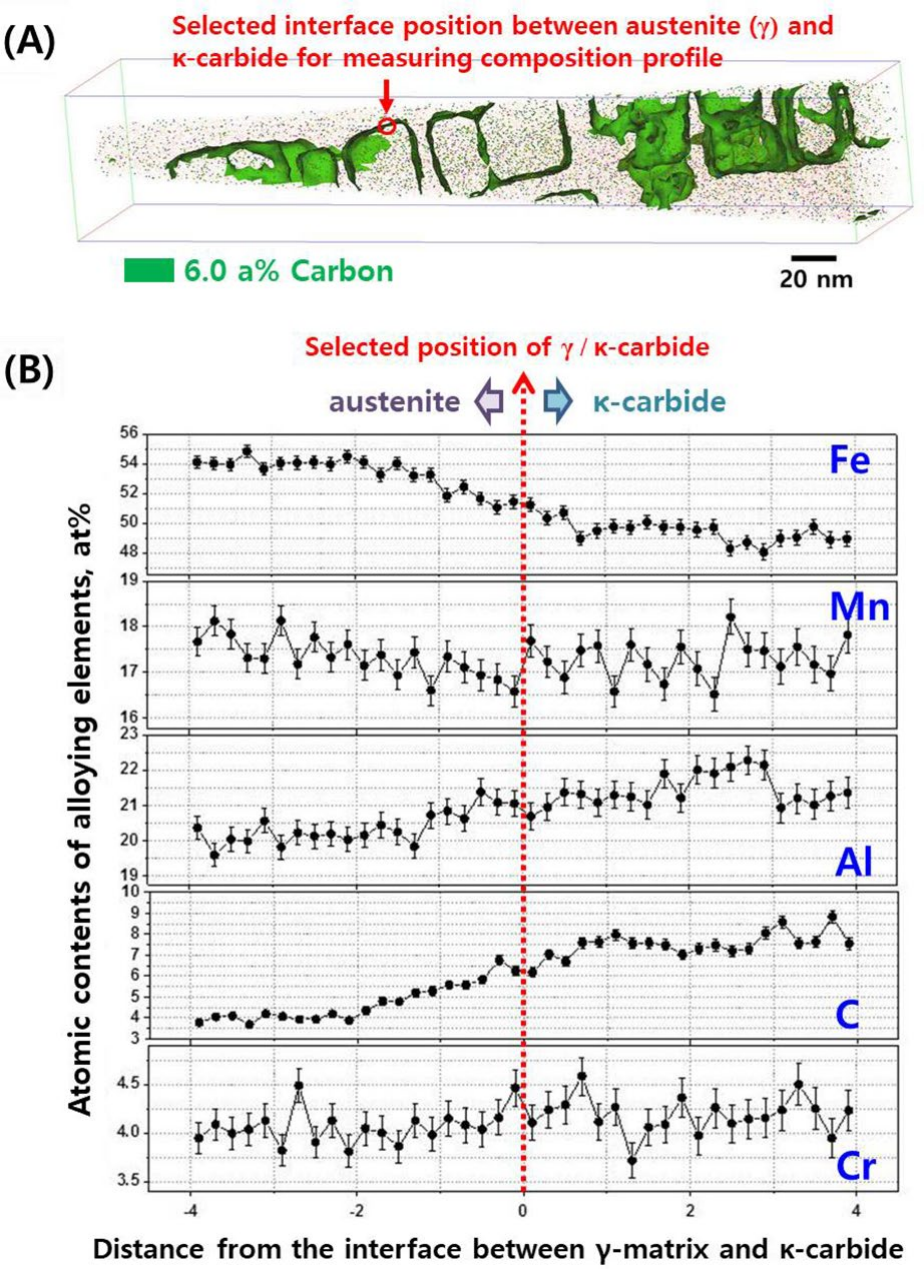
Atom probe tomography map and composition: (**A**) Atom probe tomography results visualized C (green) using 6.0 atomic % C iso-concentration surfaces for the LWSS1 alloy (Fe–20Mn–11.5Al–1.5C–5Cr), and (**B**) composition profiles across a κ-carbide interface (chosen as a concentration surface to highlight carbon-enriched regions) in the LWSS1 alloy.

The atomic-scale elemental composition of the ordered κ-carbides in the austenite was characterized by atom probe tomography (APT)^38^. Figure 5A,B present a 3-D reconstructed APT map of carbon in the new alloy LWSS1 (Fe–20Mn–11.5Al–1.5C–5Cr alloy), showing its distribution across the κ-carbide. The κ-carbides were discriminated from the austenite matrix by the 6 at% C iso-concentration surface^38^. The κ-carbides showed a typical cubic shape with sizes of approximately 30–40 nm. Figure 5B indicates that the κ-carbide was enriched in Al and C. Meanwhile, an atomic ratio of Cr/Fe for the first-principles calculations in Fig. 4 was assumed as 1/6, whereas measured ratio of Cr/Fe in Fig. 5 was approximately 1/12. This difference happened because we assumed that one Cr atom replaces one Fe atom in all unit cells (Fig. 4), while it was found that one Cr atom existed in one Fe position for every two unit cells in real state (Fig. 5). This different number of Cr atom per unit cell between simulation and experiment can affect the absolute values of formation (Fig. 4B) and strain energies (Fig. 4C) of κ-carbide, however, it is conceivable that the effect of Cr addition on formation and strain energies of κ-carbide must not be changed according to the number of Cr atom per unit cell, i.e., the addition of Cr increases the formation and strain energies of κ-carbide in proportion to number of Cr atom per unit cell and thereby suppresses the precipitation of κ-carbide.

**Figure 6:**
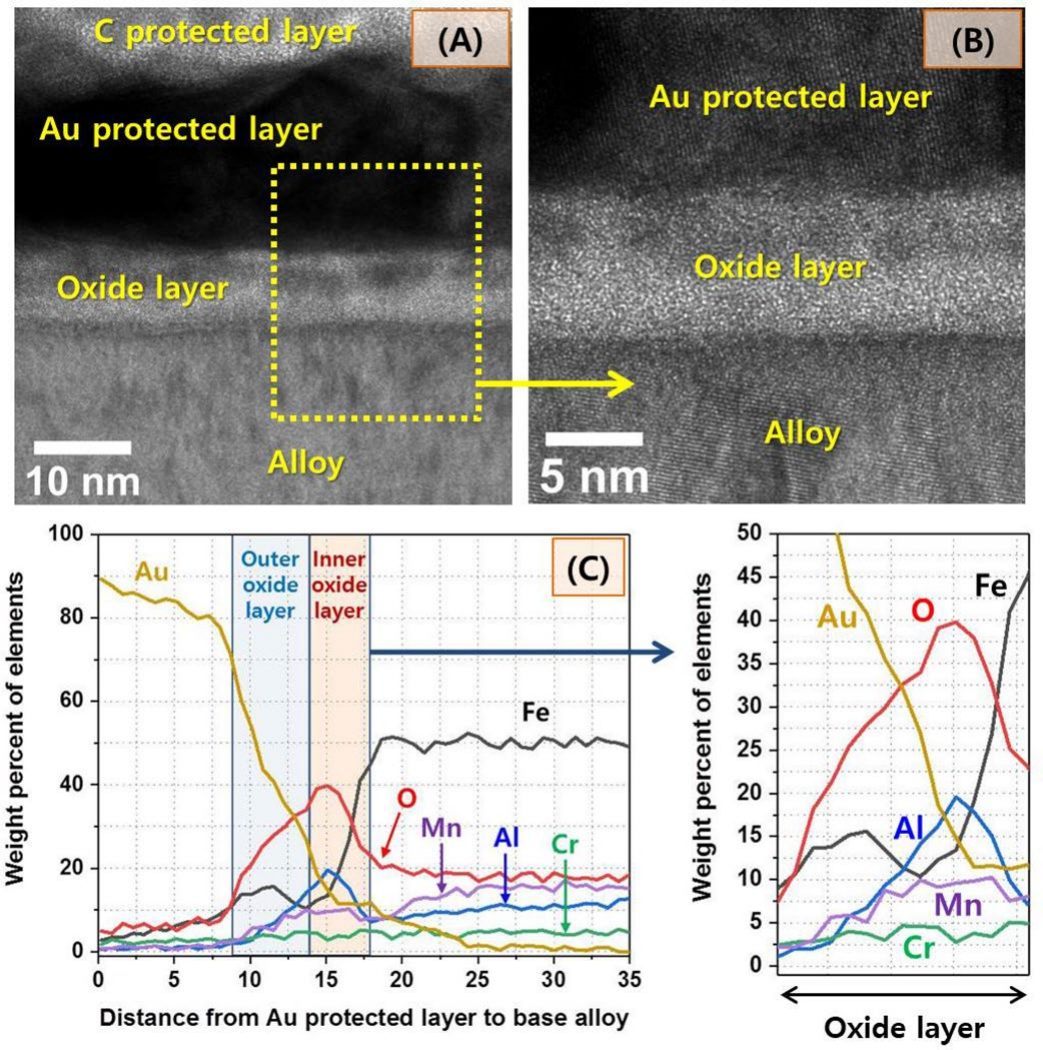
(**A**,**B**) TEM micrographs of the oxide layer on the surface of the LWSS1 (Fe–20Mn–11.5Al–1.5C–5Cr) sample and (**C**) composition profile of the oxide layer.

Next, we examined the microstructure of the surface of the new alloy LWSS1, with respect to its corrosion resistance with the aim to compete with the established expensive Ni and/ or Cr rich stainless steels. Figure 6A,B present TEM cross-sectional images through the surface, showing an oxide layer covering the surface. As shown in Fig. 6C, the oxide layer consists of double layers composed of an Fe-enriched outer layer of 3–4 nm and an Al-enriched inner layer of 4–5 nm. This result indicates that the high amount of Al included in the developed alloys produced a dense Al-enriched protective oxide layer on the surface, improving the corrosion resistance^26–28^.

**Figure 7:**
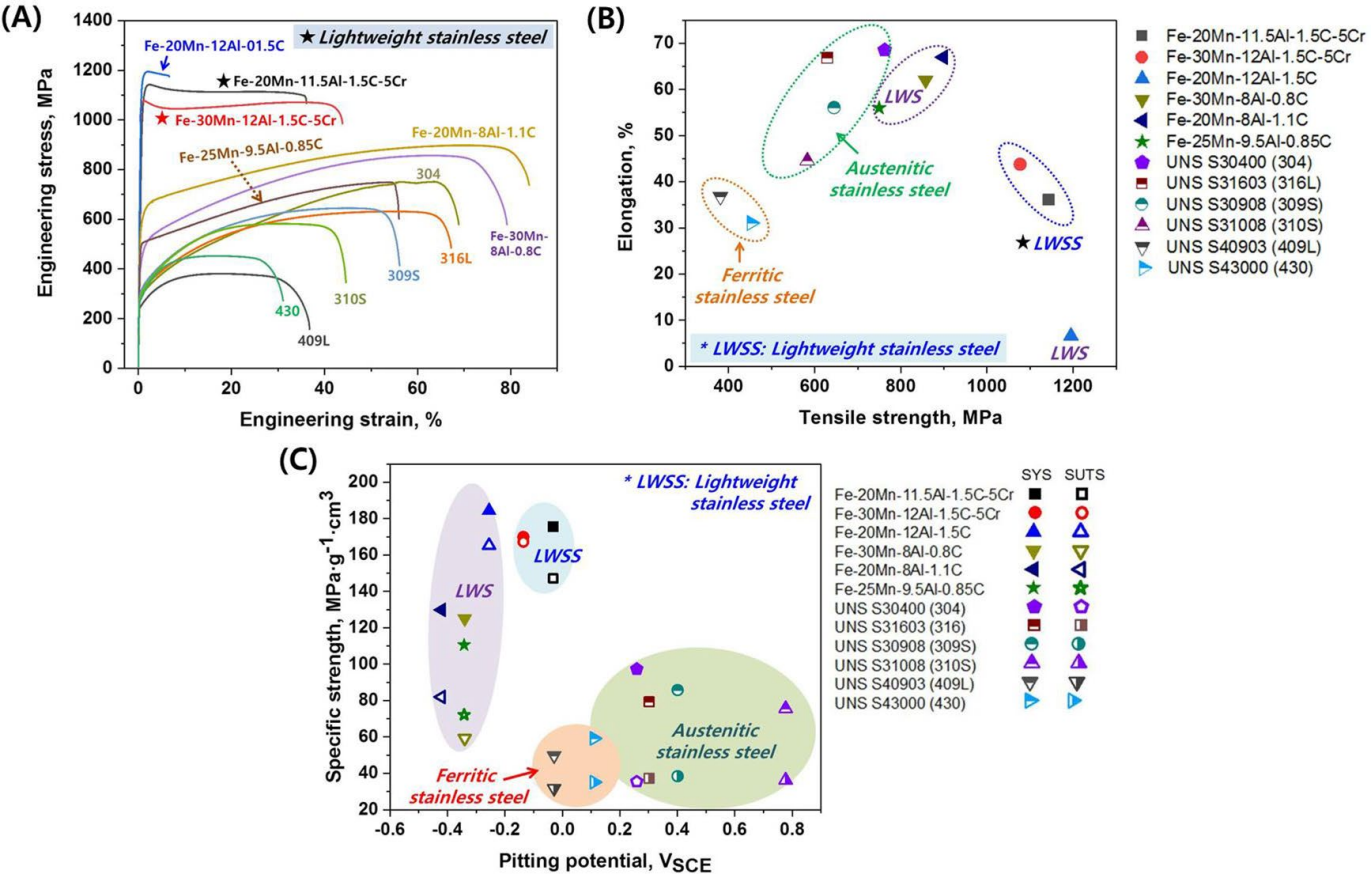
Comparison of the tensile properties and pitting corrosion resistance of the lightweight stainless steels to those of conventional lightweight steels and commercial stainless steels: (**A**) engineering stress–strain curves, (**B**) elongation versus tensile strength, and (**C**) specific strength versus pitting potential. The raw data for the tensile test and electrochemical test results are given in Fig. 8 and Table 3.

**Figure 8:**
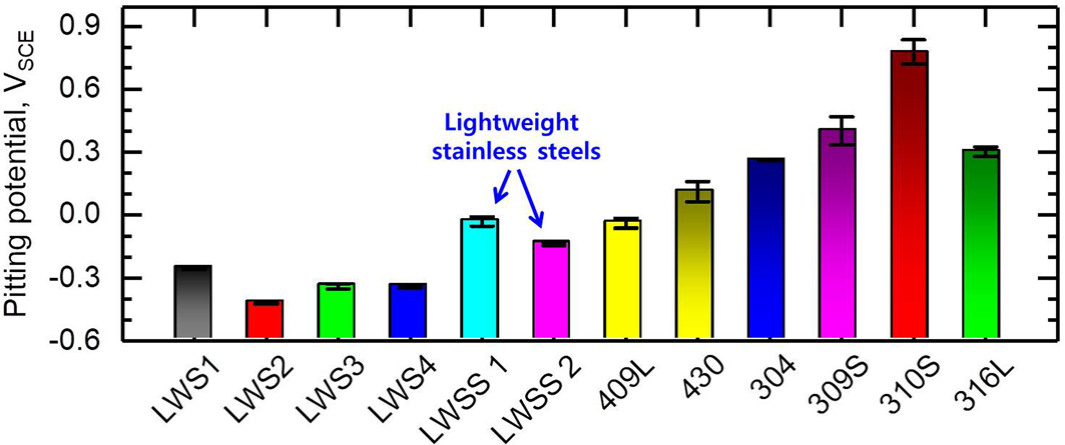
Pitting potentials of the lightweight stainless steels alloys and reference steels.

In order to evaluate the mechanical and corrosion properties of the developed LWSS alloys, we carried out tensile and potentiodynamic tests and compared the results to those of conventional austenitic lightweight steels and commercial stainless steels (see the chemical compositions listed in Table 2). Figure 7A,B show the results of the tensile tests conducted at room temperature. The newly developed LWSS alloys exhibit a balanced strength and ductility: Compared to commercial stainless steels and conventional austenitic lightweight steels, the LWSS alloys have both ultra-high strength (> 1GPa) and high tensile ductility (elongation > 35%). This result is important since in Cr-free alloys the addition of such large amounts of Al and C to lightweight steels (LWS1 in Fig. 7A) was shown before to cause significant loss of ductility with increasing strength, through formation of brittle intergranular κ-carbides, Fig. 2A, while the here presented new LWSS alloys do not suffer from this type of phase precipitated at the grain boundaries. As shown by the first principles simulations the absence of the interfacial brittle κ-phase is achieved through the Cr addition, enabling an austenite matrix strengthened only by fine ordered κ-carbides, leading to the observed excellent balance of strength and ductility in the new LWSS alloys, Figs. 2E and 4. With that the LWSS alloys exhibit a much higher specific strength than the commercial stainless steels owing to a synergistic effect between their ultra-high strength (Fig. 7B) and low mass density (Table 3). Finally, Fig. 7C shows the specific strength versus the pitting corrosion potential for the developed LWSS alloys relative to conventional lightweight steels and commercial stainless steels. The data show that the new LWSS alloys show a comparable resistance to pitting corrosion as FeCr-based ferritic stainless steels, as shown in Figs. 7C and 8.

**Table 2:** Chemical compositions of commercial stainless steels.

Alloys		Chemical composition, wt%									
		C	Mn	Cr	Ni	Mo	Cu	V	Nb	Ti	Fe
Austenitic stainless steel	304 (UNS S30400)	0.04	0.99	18.43	7.30	0.12	0.26	0.110	0.015	0.018	Bal
	316 (UNS S31603)	0.02	0.97	16.58	9.17	1.97	0.22	0.100	0.019	0.025	Bal
	309S (UNS S30908)	0.06	0.90	19.42	11.24	–	–	0.055	0.015	0.010	Bal
	301S (UNS S31008)	0.07	1.23	25.44	18.55	0.18	0.05	0.057	0.010	0.007	Bal
Ferritic stainless steel	409L (UNS S40903)	0.02	0.22	11.65	–	–	–	0.057	0.010	0.186	Bal
	430 (UNS S43000)	0.04	0.34	16.46	–	–	–	0.100	0.006	0.004	Bal

**Table 3:** The Raw tensile test results for the LWSS alloys and reference steels.

Alloys		Density, g/cm^3^	Results of tensile tests				
			YS, MPa	TS, MPa	El. %	Specific yield strength (SYS), MPa g^−1^ cm^3^	Specific ultimate tensile strength (SUTS), MPa g^−1^ cm^3^
Lightweight stainless steels (LWSS)	LWSS1	6.51	958	1,143	36.1	147.2	175.6
	LWSS2	6.34	1,060	1,077	43.8	167.2	169.9
Conventional austenitic lightweight steels (LWS)	LWS1	6.48	1,071	1,195	6.6	165.3	184.4
	LWS2	6.86	406	857	62.0	59.3	125.0
	LWS3	6.92	567	898	67.0	81.9	129.8
	LWS4	6.78	489	749	56.0	72.2	110.5
Austenitic stainless steel	304	7.84	278	762	68.5	35.5	97.3
	316	7.93	295	629	66.9	37.3	79.3
	309S	7.52	289	645	56.1	38.4	85.8
	310S	7.71	280	583	44.6	36.3	75.6
Ferritic stainless steel	409L	7.65	243	381	36.8	31.8	49.8
	430	7.65	269	453	31.1	35.2	59.2

In summary we developed here an entirely new class of sustainable, lightweight, corrosion resistant, high strength and plastically compliant, low-price alloy class to compete with established stainless steels that were invented more than 100 years ago. The developed alloys are based on the Fe–(20–30)Mn–(11.5–12.0)Al–1.5C–5Cr (wt%) system and are strengthened by fine κ-carbide. Alloying with Cr improves the ductility without a noticeable loss in strength, by suppressing the precipitation of intergranular κ-carbide and thereby stabilizing the austenite matrix. The formation of a protective Al-rich oxide film covering the surface enhances the alloys preeminent resistance to pitting corrosion similar to ferritic stainless steels. Therefore, the new alloy class has the potential to replace commercial stainless steels owing to its higher strength at similar formability and 17% lower mass density, enabling the innovative design of structural parts demanding lightweight, corrosion resistant, and high-strength.

## Materials and methods

### Sample preparation

Here, we developed a new family of ultra-low-density stainless steels (LWSS alloys: Fe–20Mn–11.5Al–1.5C–5Cr alloy and Fe–30Mn–12Al–15C–5Cr alloy) and compared them to conventional lightweight steels [LWS alloys: Fe–(20–30)Mn–(8–12)Al–(0.8–1.5)C] and commercial stainless steels. For the stainless steel, we purchased and tested commercial austenitic, ferritic, and martensitic stainless steels. For the LWS and LWSS alloys, we prepared ingots using a commercial vacuum-induction melting (VIM) furnace. The ingots were initially reheated for 2 h at 1,100 °C and then hot-rolled into plate samples with a thickness of 7 mm. Finally, the hot-rolled samples were solution-treated for 2 h at 1,050 °C and water-quenched.

### Microstructural characterization

The microstructures of specimens after the solution-treatment were examined with scanning electron microscopy (SEM) and transmission electron microscopy (TEM) analyses. For SEM analyses, the specimens were mechanically polished and then chemically etched in a mixed solution of ethanol (100 ml) and nitric acid (40 ml). Thin foil specimens for the TEM analyses were prepared by twin-jet electrolytic polishing at 20 V and 200 mA with a mixed solution of 10% perchloric acid and 90% methanol at − 25 °C^39^.

The distribution of alloying elements across the κ-carbide within the austenite matrix was analyzed by atom probe tomography (APT) with a CAMECA LEAP 4000X HR^34^. Tip samples for the analyses were prepared using a focused ion beam (FIB) equipment^34^. The tips were held in a vacuum of 8.5 × 10^–12^ Torr at − 238.75 °C (34.4 K), after which they were field-evaporated at an evaporation rate of 0.5% with 355 nm UV laser at a laser power of 100 pJ and a pulse repetition rate of 125 kHz. The data of APT was dissected through Interactive Visualization and Analysis Software (IVAS 3.8.4) of CAMECA instruments. The proximity diagrams from selected interfaces were analyzed using the standard analysis method of IVAS software. The Al contents can be over-estimated due to the mass-to-charge spectrum overlap of Al^1+^ and Fe^2+^ at 27 Da and the ratio of contents between Al and Fe at 27 Da was 85.2 and 14.8, respectively.

### Tensile and electrochemical tests

The stress–strain responses of the samples were measured by tensile testing at a nominal strain rate of 1.33 × 10^–3^ s^–1^. For the tensile tests, sub-size tensile specimens with dog-bone shapes were machined according to the ASTM A370 standard; i.e., the length of the gauge was 25 mm, the width of the reduced section was 6.25 mm, and the overall length of the specimen was 100 mm.

The pitting corrosion resistance of the LWS and LWSS alloys listed in Table 2 was evaluated through potentiodynamic polarization tests in a 0.6 M (3.5 wt%) NaCl solution at 20 ± 1 °C by measuring the pitting potential. The pitting potentials of the alloys in Table 2 were compared with those of the commercial stainless steels given in Table 3. The polarization tests were performed using a 3-electrode cell with a saturated calomel reference electrode (SCE) and Pt counter electrode; the alloy specimen served as the working electrode and was connected to a potentiostat (Reference 600, GAMRY Instruments). The specimen (W10 mm × L10 mm × T2 mm) for the working electrode was connected to copper wire and cold mounted in epoxy resin. The mounted specimen was wet ground using SiC emery paper up to 2000-grit. The exposed electrode area was controlled to 0.2 cm^2^ using electroplating tape. During the polarization tests, the working electrode was anodically polarized from − 0.1 V versus the corrosion potential to the pitting potential with a potential sweep rate of 1 mV s^−1^. Before the polarization tests, the specimens were cathodically polarized by applying − 1 V_SCE_ for 300 s to remove the air-formed oxide film.

### First-principles calculations

The effects of Cr on the precipitation of κ-carbide were calculated through first-principles calculations within the framework of density functional theory (DFT). The single-electron equations were solved using the pseudo-potential method implemented in the Vienna Ab-Initio Simulation Package (VASP)^30,31^. All of the self-consistent calculations were performed by means of the generalized gradient approximation (GGA) for the exchange–correlation potential^32^. The calculations were carried out based on the unit cell of κ-carbide with one formula unit of Fe_3_AlC. Lattice parameters and internal atomic positions were fully relaxed toward equilibrium until the forces fell below 10^–3^ eV/A^33^. The formation energy and interfacial energy were calculated for Cr substitution structures in the κ-carbide to evaluate the effects of Cr on the precipitation of κ-carbide. All simulations have been conducted for 0 K.

## Data Availability

The datasets generated during and/or analysed during the current study are available from the corresponding author on reasonable request.
